# Characterization of Feeding Behaviors, Appetite Regulation and Growth Performance of All-Female (*cyp17a1*+/−;XX Genotype) Common Carp (*Cyprinus carpio*)

**DOI:** 10.3390/ijms252312517

**Published:** 2024-11-21

**Authors:** Xuehui Li, Qingqing Zou, Xuebo Liu, Qiyong Lou, Xia Jin, Jiangyan He, Zhan Yin, Gang Zhai, Ming Duan, Guanghui Chen

**Affiliations:** 1 State Key Laboratory of Freshwater Ecology and Biotechnology, Institute of Hydrobiology, Chinese Academy of Sciences, Wuhan 430072, China; lixuehui22@mails.ucas.ac.cn (X.L.); zouqingqing@ihb.ac.cn (Q.Z.); louqiyong@ihb.ac.cn (Q.L.); jinxia@ihb.ac.cn (X.J.); jyhe@ihb.ac.cn (J.H.); zyin@ihb.ac.cn (Z.Y.); zhaigang@ihb.ac.cn (G.Z.); 2 College of Advanced Agricultural Sciences, University of Chinese Academy of Sciences, Beijing 100101, China; 3 College of Marine Sciences, South China Agricultural University, Guangzhou 510642, China; 20233140038@stu.scau.edu.cn; 4 Hubei Hongshan Laboratory, Huazhong Agriculture University, Wuhan 430070, China; 5 The Innovative Academy for Seed Design, Chinese Academy of Sciences, Beijing 100101, China

**Keywords:** genome editing, all-female carp, feeding behavior, appetite regulation, growth performance

## Abstract

Genome editing has the potential to improve growth and traits of aquatic animals. Assessment of the feeding habits of the genetically modified farmed fish is necessary, as this is closely related to the assessment of their growth performance, which is one of the most important economic traits. Previously, we developed a novel strategy to produce all-female (AF) common carp (*cyp17a1*+/−;XX genotype) with genome editing, which exhibited a growth advantage compared to the control carp (including control male and female carp). However, the feeding behavior related to the growth performance of wild-type control and AF common carp remains elusive. The results of feeding and swimming behaviors showed that AF common carp exhibited a faster feeding activities and more active swimming activities, which probably enhanced its growth performance. Brain gene expression analysis revealed AF common carp had a significant upregulation of the orexigenic factors gene expression levels in the fed state, which would further promote the growth of AF carp. Here, AF carp exhibited higher growth performance with higher growth hormone (*gh*) gene expression. This study provided insight into the growth performance, feeding behaviors and appetite regulation of the genetically modified AF carp and the assessment of feeding behaviors in other genetically modified farmed fish.

## 1. Introduction

Numerous fish species exhibit obvious sexual dimorphism in growth, such as common carp (*Cyprinus carpio*), rainbow trout (*Oncorhynchus mykiss*), bastard halibut (*Paralichthys olivaceus*), with females being larger than males; and tilapia (*Oreochromis niloticus*), channel catfish (*Ictalurus punctatus*) and yellow catfish (*Pelteobagrus fulvidraco*), with males being larger than females [1]. The exploitation of sexual dimorphism in fish growth for the creation of single-sex populations represents a significant avenue of development for the aquaculture industry, with the potential to enhance and improve production and the value of aquaculture.

Common carp is one of the most important fish species, which has been cultivated widely in more than 100 countries [2]. With the continuous improvement of human living standards, the per capita intake of fish protein continues to rise [3]. Approximately 97% of global carp production comes from aquaculture [4]. However, global carp production has declined from 8% in 1990 to 4.6% in 2020 [5]. China is the world’s leading producer of common carp, and its production of carp has fallen from a peak of 3.5 million tons in 2016 to 2.83 million tons in 2021 [6,7]. Therefore, there is a high demand for genetic modification to enhance its qualities and economic value and to facilitate its use as a biological model. Because of its comparatively long maturation period, it is difficult to carry out genetic studies and breeding of common carp [2].

Common carp exhibits sexual dimorphism in growth and XX/XY type genetic sex determination, with females growing 20% faster than males [8]. Therefore, monosexual fish production is desirable for common carp breeding. Previous studies have revealed that genotypic sex, such as neomale or neofemale fish, could be reversed through the application of exogenous sex steroid hormones [9,10,11]. Hence, the hormonal induction of sex reversal is a broadly used technique in the fisheries industry for the large-scale production of monosexual fish populations. However, the production and maintenance of hormone-induced sex reversal is unstable and laborious, and it requires a comparatively long rearing period [9]. In recent years, gene editing technologies have emerged as a promising means to deal with this problem and improve the production of common carp. Most recently, the CRISPR/Cas9 technique was used by us to knock out a key testosterone synthesis gene *cyp17a1* in Yellow River carp [12]. Interestingly, the gonads of *cyp17a1*−/−;XX carp developed into testes, which presented a typical neomale phenotype with normal spermatogenesis [12]. Sperm from neomale carp (*cyp17a1*−/−;XX) were artificially fertilized with the eggs from wild-type (WT) females (*cyp17a1*+/+;XX), and their offspring genotype was identified as *cyp17a1*+/−;XX (termed as AF common carp) by examination using our male-specific markers. The body weight of the AF carp group showed a prominent improvement of 22.58% compared to the control group [13]. Studies have shown that multitudinous physiological factors can influence fish growth, including feeding behaviors, genetic background, nutritional status, endocrine hormone regulation, age and development stages [14,15,16,17]. However, knowledge regarding the regulation of feeding behavior of AF carp is still limited.

Feeding behavior affects the ability of fish to compete for food resources, which is critical for survival, competition and growth [18]. Fish have been found to display individual differences in behavior that lead to a variety of ecologically important outcomes, including ingestion [19,20]. Studies have suggested that fish with higher speed or shorter initial capture times have a greater capacity to capture pellets [21,22]. Therefore, individual differences in fish feeding behaviors may be a factor leading to the differences in growth. Although the AF carp showed the advantage of rapid growth, the behavioral differences between individuals in terms of feeding capacity are still poorly understood.

Feeding behavior in fish is influenced by a multitude of environmental factors and complex homeostatic mechanisms [23]. Similar to mammals, the physiological regulation of appetite and ingestion consists of a complex integration of peripheral and central signals by the brain [24]. Peripheral endocrine and metabolic factors convey information about the nutritional status to the brain, and subsequently the brain processes the information and initiates the release of neuropeptides from neurons to control feeding behavior [25]. Among these factors, orexigenic factors that stimulate food intake factors mediate the appetite-regulatory effects, including GH, agouti-related neuropeptide 1 (AGRP1), neuropeptide Y (NPY) and orexin (HCRT) [26]. However, the precise physiological role of each factor is not fully understood. Therefore, the research strategies for feeding behavior in carp remain to be further explored.

As an important freshwater species in aquaculture, common carp is valued for its high-quality meat and potential for rapid growth, with obvious sexual dimorphism in growth. Therefore, it is a pivotal fitness parameter to evaluate and explore sex differences in growth performance, appetite regulation and feeding behaviors in carp. Here, we compared the growth performance, appetite regulation and feeding behaviors of AF and wild-type control carps, which would provide better knowledge of feeding behavior in fish. This will help to optimize diets and feeding protocols in fish and ultimately enhance the growth rates of numerous aquaculture species.

## 2. Results

### 2.1. AF Carp Exhibited Faster Feeding Activities

**Figure 1:**
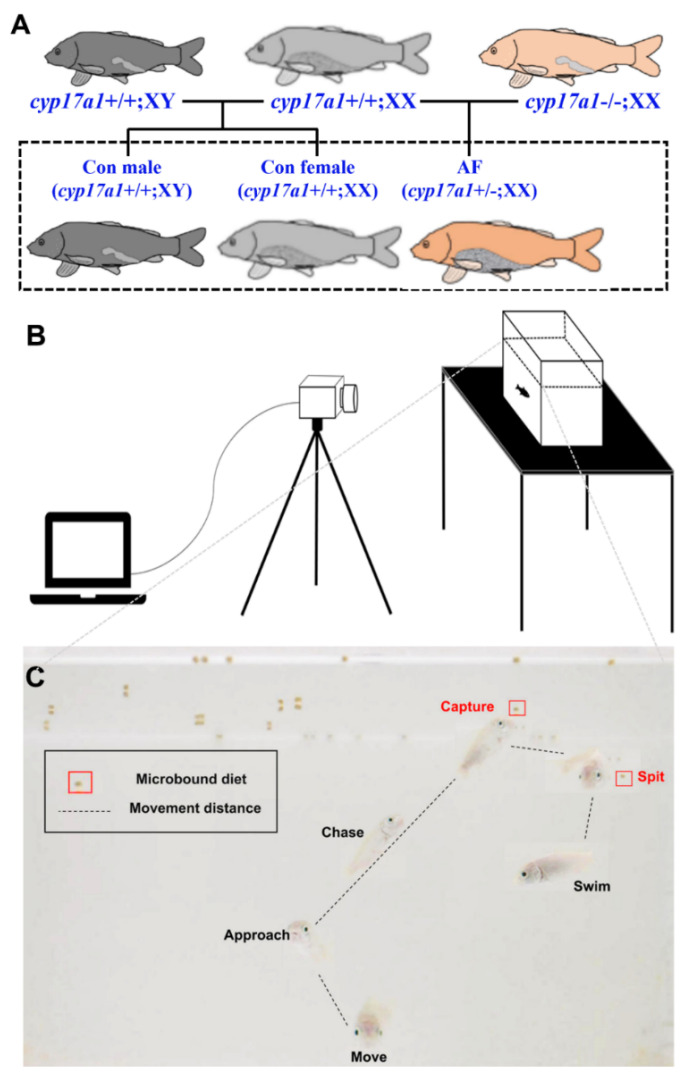
Feeding behavior analysis. (**A**) The procedure process of AF and control common carps. (**B**) The schematic diagram of the feeding behavior device. (**C**) The diagram of a complete feeding process of AF and control common carp.

The complete feeding process of carp consists of a series of actions, including movement, approach, chase, capture, spitting and swimming (Figure 1C and Movie S1). Once the diet was located, the carp changed direction and chased the diet at a high speed, captured it and then quickly swam away, making repeated attempts. The carp swallowed food quickly after chewing through their pharynx.

During the experimental period, we measured the average body weight and weight gain rate of control female, control male and AF common carp (Figure S1). They did not show remarkable differences between control female, control male and AF common carp (*p* > 0.05). In terms of weight gain, AF carp exhibited the higher weight gain on day 45 (*p* < 0.05), but they did not show remarkable differences between control female and control male carp (*p* > 0.05).

**Figure 2:**
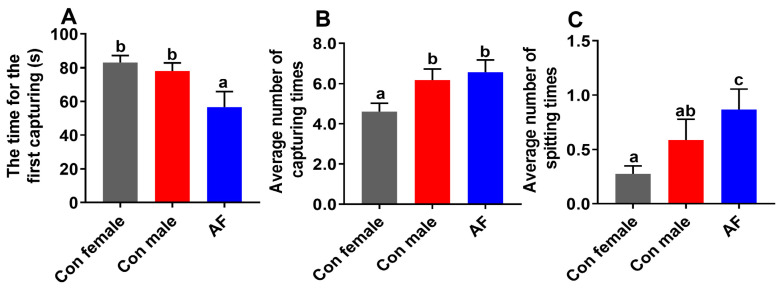
The results of feeding behavior analysis. (**A**) The time of the first capture. (**B**) Average number of capture times. (**C**) Average number of spitting times. Values are means ± SEM (n = 3). Letters indicate significant differences among the different groups. The time of the first capture (s): the time interval from the beginning of the microbound diet supplementation to the first capture by the experimental fish. The frequency of capture and spitting were manually marked.

After the addition of the diets, the latency time of the first capture for AF carp was shorter than that for control females and control males (*p* < 0.05), but there were no remarkable differences between control females and control males (*p* > 0.05) (Figure 2A). The average number of capture times for control female carp was less than that for AF carp and control males (*p* < 0.05), but there were no remarkable differences between AF carp and control males (*p* > 0.05) (Figure 2B). The average number of spitting times for control female carp was less than that for AF carp (*p* < 0.05), but there were no remarkable differences between control females and control males (*p* > 0.05) (Figure 2C).

**Figure 3:**
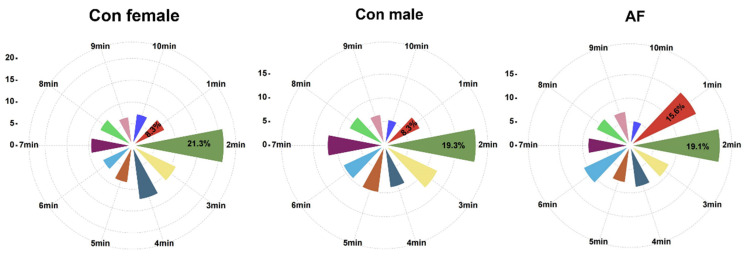
Rose diagrams of capture diets events per minute. Percentage meant capture or spitting events in the first minute accounted for the total events within 10 min.

Within 1 min after the diet was added, the feeding activities of AF carp were extremely frequent, accounting for 15.6% of the total feeding time, which was higher than those of control female and control male carp (Figure 3).

### 2.2. Gene Expression Involved in Orexigenic Factors

**Figure 4:**
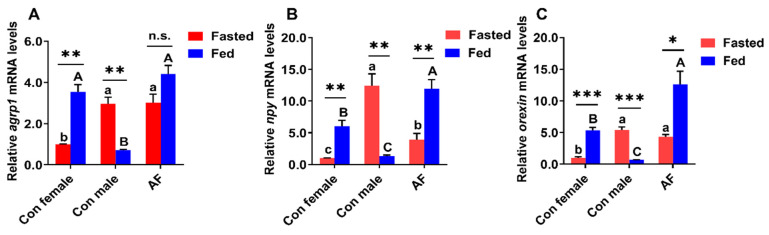
Gene expression involved in orexigenic factors of AF and control common carps in fasted and fed sates. (**A**) *agrp1* mRNA levels. (**B**) *npy* mRNA levels. (**C**) *orexin* mRNA levels. Values are means ± SEM (n = 3). The lowercase letters indicate significant differences among the fasted groups. The uppercase letters indicate significant differences among the fed groups. *** *p* < 0.001. ** *p* < 0.01. * *p* < 0.05. n.s., no significance.

To determine the mechanism of the faster growth performance of AF carp, we analyzed the expression of genes involved in orexigenic factors in the brain of fish in the fasted and fed states. In the fasted state, the mRNA level of *agrp1* in the control female carp was the lowest and lower than those in the AF and control male carps (Figure 4A) (*p* < 0.05); however, in the fed state, the mRNA levels of *agrp1* in the control male carp was the lowest and significantly lower than those in the AF and control female carps (*p* < 0.05). Compared to the fasted fish, the fed control female carp possessed higher *agrp1* expression levels, but the fed control male carp possessed lower *agrp1* expression level (*p* < 0.05), and the *agrp1* gene expression of the fed AF carp did not show prominent differences (*p* > 0.05).

In the fasted state, *npy* gene expression was lowest in the control female carp and lower than those in control male and AF carps (Figure 4B) (*p* < 0.05); however, in the fed state, *npy* gene expression was highest in the AF carp and significantly higher than those in control females and control males (*p* < 0.05). Compared to the fasted fish, the fed AF and control female carps possessed higher *npy* expression levels, but the control male carp possessed lower *npy* expression level (*p* < 0.05).

In the fasted state, the mRNA level of *orexin* was the lowest in the control females and lower than those in control males and AF carp (Figure 4C) (*p* < 0.05); however, in the fed state, the mRNA level of *orexin* was the highest in the AF carp and significantly higher than those in control females and control males (*p* < 0.05). Compared to the fasted fish, the fed AF and control female carp possessed higher *orexin* expression levels, but the fed control male carp possessed lower *orexin* expression levels (*p* < 0.05).

### 2.3. AF Carp Exhibited More Active Swimming Activities

**Figure 5:**
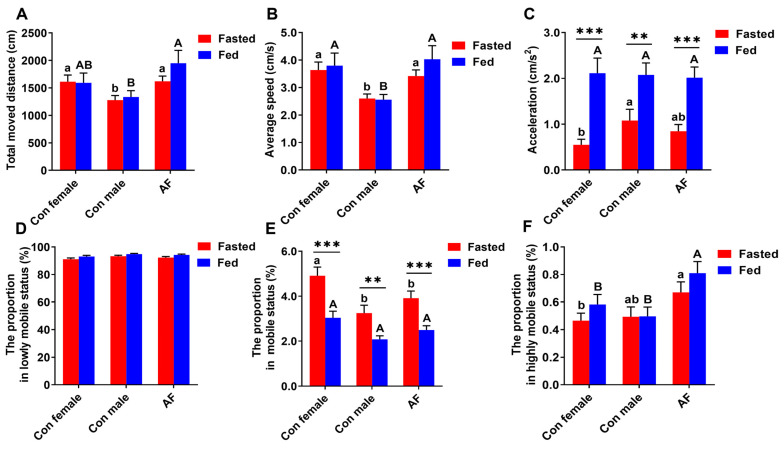
Swimming activity levels of AF and control common carps in fasted and fed sates. (**A**) Total moved distance. (**B**) Average speed. (**C**) Accelerated speed. (**D**) The proportion in lowly mobile state. (**E**) The proportion in mobile state. (**F**) The proportion in highly mobile state Values are means ± SEM (n = 3). The lowercase letters indicate significant differences among the fasted groups. The uppercase letters indicate significant differences among the fed groups. *** *p* < 0.001. ** *p* < 0.01. Total moved distance (cm): total distance travelled by the geometric center point of experimental fish. Average speed (cm/s): average speed of locomotion of the geometric center point of experimental fish. Acceleration (cm/s^2^): defined as acceleration at which the central point of the fish moves. Lowly mobile was below 20% mobility; mobile was higher than 20% mobility and lower than 80% mobility; highly mobile was higher than 80% mobility.

In the fasted state, the total moved distance for control male carp was shorter than that for AF carp and control female carp (*p* < 0.05) (Figure 5A); in the fed state, the total moved distance for control male carp was the shortest and was shorter than that for AF carp (*p* < 0.05). Compared to the fasted fish, the total moved distance for the fed fish did not show significant differences between control female, control male and AF carp (*p* > 0.05).

In the fasted and fed states, the average speed for control male carp was slower than those for AF carp and control female carp (*p* < 0.05) (Figure 5B). Compared to the fasted fish, the total moved distance for fed fish did not show significant differences between control female, control male and AF common carps (*p* > 0.05).

In the fasted state, the acceleration for control male carp was faster than that for control female carp (*p* < 0.05) (Figure 5C); in the fed state, acceleration did not show significant differences between control female, control male and AF common carps (*p* > 0.05). Compared to the fasted fish, acceleration for fed fish was significantly promoted for control female, control male and AF common carps (*p* < 0.05).

The mobility state was divided into three items (lowly mobile, mobile and highly mobile according to the changes of carp movement. As for the percentage of lowly mobile state, whether in the fasted state or the fed state, they did not show significant differences for control female, control male and AF common carps (*p* > 0.05) (Figure 5D).

In the fasted state, the percentage of mobile states was the highest for control female carp and markedly higher than that for AF carp and control female carp (*p* < 0.05) (Figure 5E); however, the percentage of mobile states in the fed fish did not show significant differences for control female, control male and AF common carps (*p* > 0.05). Compared to the fasted fish, the percentage of mobile states for the fed fish was reduced for AF carp and control female and male carps (*p* < 0.05).

In the fasted state, the percentage of highly mobile states was the highest for AF carp and higher than that for control female carp (*p* < 0.05) (Figure 5F); in the fed state, the percentage of highly mobile states was the highest for AF carp and higher than that for control female carp and control male carp (*p* < 0.05). Compared to the fasted fish, the percentage of highly mobile states for the fed fish did not show significant differences between control female, control male and AF common carps (*p* > 0.05).

**Figure 6:**
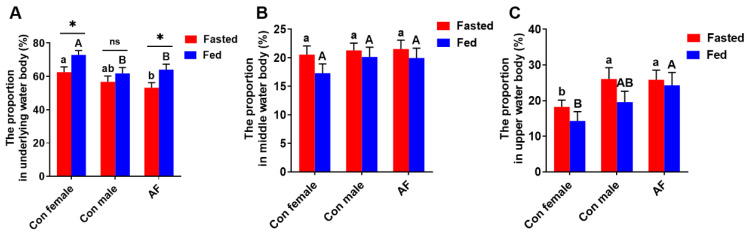
The water layer distribution of AF and control common carps in fasted and fed sates. (**A**) The proportion in underlying water layer. (**B**) The proportion in middle water layer. (**C**) The proportion in upper water layer. Values are means ± SEM (n = 3). The lowercase letters indicate significant differences among the fasted groups. The uppercase letters indicate significant differences among the fed groups. * *p* < 0.05. ns, no significance. Duration in different water layers was defined as cumulative duration of fish in different water layers (the study area was divided equally into three aquifers: upper, middle and lower).

In the fasted state, the proportion of time spent in the underlying water body was the most for control female carp and markedly more than that for AF carp (*p* < 0.05) (Figure 6A); in the fed state, the proportion of time spent in the underlying water body was the highest for female carp and markedly higher than that for AF and control male carps (*p* < 0.05). Compared to the fasted fish, the proportion of time spent in the underlying water body was higher for fed fish than those for control female and AF common carps (*p* < 0.05).

The proportion of time spent in the middle water body, whether in the fasted state or in the fed state, did not show significant differences for control female, control male and AF common carps (*p* > 0.05) (Figure 6B).

In the fasted state, the proportion of time spent in the upper water body for control female carp was the shortest and significantly shorter than those for AF carp and control male carps (*p* < 0.05) (Figure 6C); in the fed state, the proportion of time spent in the upper water body for AF was the greatest and significantly greater than that for control female carp (*p* < 0.05). Compared to the fasted fish, the proportion of time spent in the upper water body for fed fish did not show significant differences for control female, control male and AF carps (*p* > 0.05).

### 2.4. AF Carp Exhibited Higher Growth Performance

**Figure 7:**
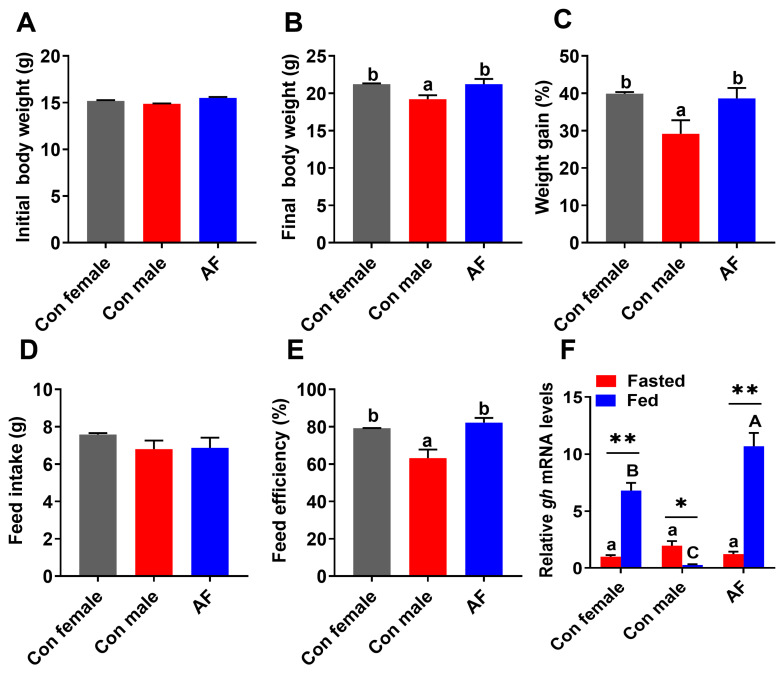
The growth performance of AF and control common carps. (**A**) Initial body weight. (**B**) Final body weight. (**C**) Weight gain. (**D**) Feed intake. (**E**) Feed efficiency. (**F**) *gh* gene expression. Values are means ± SEM (n = 3). The lowercase letters indicate significant differences among the fasted groups. The uppercase letters indicate significant differences among the fed groups. ** *p* < 0.01. * *p* < 0.05.

During the experimental period, average body weight and weight gain (WG), feed intake (FI) and feed efficiency (FE) of control female, control male and AF carps were measured (Figure 7A–E). AF and control female carp had higher body weight and weight gain than control male carp (*p* < 0.05). There were no notable differences in FI among all groups (*p* > 0.05). The FE of the control male, control female and AF carps were 63.17%, 79.22% and 82.17%, respectively. The average of FE of the control male and female carp was 71.195%, whereas that of the AF carp improved 15.4% compared to control carp.

In the fasted state, *gh* gene expression did not show significant differences among control female, control male and AF carps (Figure 7F) (*p* > 0.05); however, in the fed state, the mRNA level of *gh* of the AF carp was the highest and higher than those of control female and control male carps (*p* < 0.05). Compared to the fasted fish, the fed AF and control female carp possessed higher *gh* expression levels, but the fed control male carp possessed lower *gh* expression levels (*p* < 0.05).

### 2.5. The Relationships Between Fish Movement and Their Feeding Behaviors

**Figure 8:**
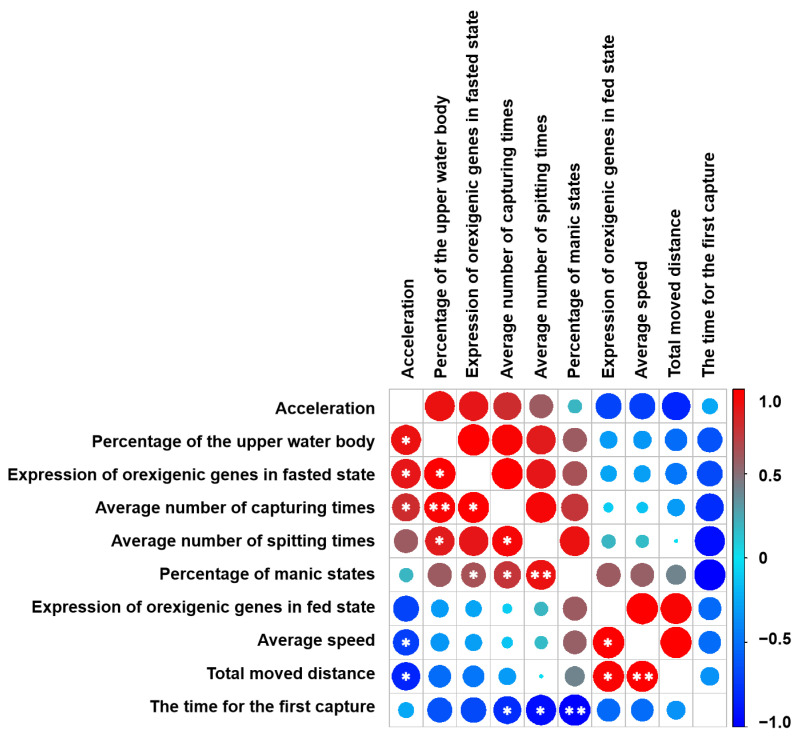
The correlation relationships among the feeding behaviors of common carp. ** *p* < 0.01. * *p* < 0.05.

The Spearman correlation was used to analyze the differences between the variables before and after the ingestion of the diets (Figure 8). There was an obvious negative correlation between the time of the first capture and the average number of capturing times, the average number of spitting times and the percentage of manic state. Results showed that the total moved distance was positively correlated with average speed and the expression of orexigenic genes in the fed state, but negatively correlated with acceleration. Average speed was negatively correlated with acceleration, but positively correlated with the expression of orexigenic genes in the fed state. The percentage of manic state was positively correlated with the average number of capturing times, the average number of spitting times and the expression of orexigenic genes in the fasted state. The average number of spitting times was positively correlated with the average number of capturing times and the percentage of time spent in the upper water body. The average number of capturing times was positively correlated with the average number of spitting times, the expression of orexigenic genes in the fasted state and the acceleration. The expression of orexigenic genes in the fasted state was positively correlated with percentage of time spent in the upper water body and acceleration. There was an obvious positive correlation between percentage of time spent in the upper water body and acceleration (*p* < 0.05).

## 3. Discussion

Common carp, one of the world’s most important freshwater fish, shows sexual dimorphism in its somatic growth [8]. As females grow significantly faster than males, the production of monosex fish is deemed to possess substantial potential in the aquaculture industry. Using genetic engineering, all-female common carp were successfully created via targeting *cyp17a1* gene manipulation [12]. In our previous study, the body weight of the AF carp group showed a significant improvement of 22.58% compared to the control carp reared in the same pond [13]. Here, the body weight and weight gain of AF and control female carps were higher than that of control males under separate rearing conditions. However, FI did not show remarkable differences among all groups. Studies have found that growth and feed intake are not correlated, but they are correlated with feed efficiency [27]. Improving feed efficiency is a key to reduce production costs and achieve productivity and sustainability for the aquaculture industry [27]. In the present study, AF carp had the highest feed efficiency. In fish, studies have pointed out that exogenous GH administration would promote food intake and feeding behavior [18,28]. GH transgenesis is known to increase growth and consequently metabolic rate in fish. Similarly, higher GH levels in transgenic salmon resulted in increased feeding motivation [29,30]. Thus, the higher *gh* expression after ingestion would promote growth in AF carp. These results suggested that AF carp, whether reared in the same pond or bred separately, exhibited faster growth performance. However, the intrinsic relationship between the greater growth of all-female carp and the nutritional quality of the fish flesh remains to be explored.

Feeding behavior affects the capability of fish to compete for food resources [18]. Therefore, it serves as an important fitness parameter for assessing the ecological impacts of AF carp. Under actual farming conditions, fish are reared in groups, often with large numbers of individuals. In the present study, the isolated individual was used to analyze feeding behaviors and facilitate data collection. Thus, some stress effects for fish could be eliminated by acclimating them 10 min before filming began [22]. The behavioral data obtained from the video (Movie S1) showed that AF carp ingested food quickly and then spat it out. During the experiment, there was such a tendency for AF carp, e.g., the shorter time for the first capture and the increased average number of capturing times. In particular, AF carp was found to be susceptible to a higher percentage of diet capturing events in the first minute from the rose diagrams, showing an overall trend of increased ability to ingest food in the laboratory than control carp. For the same size, the sexual maturity of the fish differed between males and females. This may partly explain the differences in feeding behavior observed between the various genotypes. In fish, sex steroid hormones may link sex and feeding behavior. Our previous data showed that female carp had lower androgen and higher estrogen levels than male carp, which were closely related to fish growth [22]. Testosterone and estrogen significantly affect feeding behavior and appetite [31]. Male and female fish may exhibit different feeding patterns, particularly during the breeding season when sex hormone levels fluctuate.

In general, the swimming behavior of each carp was observed to understand how each fish responded to the diet. Here, we observed that there were three states for carp, including lowly mobile, mobile and highly mobile states. Our findings indicated that the swimming behaviors of carp influenced their food consumption, and more active carp had a greater potential to ingest the diet. It has been proved that more active fish presented higher swimming performance [32]. Therefore, it was expected that the active fish would have a higher average speed and swim farther in the tank, which makes them more likely to come across and active consume the food. When comparing fasted and fed conditions, the study showed that AF carp tended to have higher overall scores for activity, distance travelled and speed. In the current laboratory situation, due to the floating feed used in the experiment, carp swimming in the upper part of a school possessed higher feeding rates, suggesting that AF carp in the upper part of the water body have a greater chance of ingesting food. Although the swimming performance of the AF carp was better than that of the control, the expected trend in relation to the feeding treatment was not found. Meanwhile, the weight gain exhibited a positive correlation with the average speed of the fish, signifying the degree of activity of the fish [21]. It is worth noting that the Spearman correlation was only used to identify correlations between the different variables observed, but the positive correlations do not correspond to cause-and-effect relationships.

Feeding behavior in fish is regulated by numerous environmental factors as well as complex homeostatic mechanisms. Appetite regulation has been suggested to play an important regulatory role in feeding behaviors of fish [33]. Not only did we observe differences in the average weight gain between different experimental carp, but we also found that there were obvious differences in orexigenic factors in the brain between individual fish. Our results showed that AF carp had higher gene expression of orexigenic factors, including *agrp1*, *nay* and *orexin*. AgRP has been shown to be a pivotal orexigenic neuropeptide in fish [34,35]. The elevated *agrp1* mRNA levels in AF carp were consistent with the role of orexigenic peptides. NPY has been proved to be one of the most potent orexigenic factors in vertebrates [25]. It was thus estimated that AF carp may have increased *npy* expression relative to the control carp, which contributed to the stimulated appetite after ingestion. Orexins are involved in the regulation of feeding physiology, as exemplified by the fact that the injections of human orexin A and B prompt feeding behavior and food consumption in fish [36]. In the fed state, the expression of orexins was significantly increased in AF carp, which in turn stimulated appetite and increased growth. In addition, the growth difference of male and female fish in mixed culture may be due to the different nutritional requirements of male and female fish. Nutrition is a crucial internal factor in fish biology and significantly impacts their growth. As a primary external signal, feed plays a key role in stimulating fish feeding behavior and growth in fish. The feed characteristics, such as feed composition and pellet size, can influence these behaviors, and the effects may vary depending on the sex of the fish. Thus, the behavioral responses of fish to feeding have been associated with feeding approach, feeding habits, feeding regularity, feeding detection mechanisms and feeding preferences [15].

In conclusion, AF carp exhibited higher growth performance, shorter capture time, higher activity scores, moved distance and speed. Taken together, these results suggest that AF carp would inherently have enhanced appetite and feeding motivation, thereby improving their ability to compete for limited food resources.

## 4. Materials and Methods

### 4.1. Fish and Experimental Conditions

The experimental fish, including the generated AF (*cyp17a1*+/−;XX) and control common carp (*cyp17a1*+/+;XY and *cyp17a1*+/+;XX), were obtained by crossing the neomale common carp (*cyp17a1*−/−;XX) or the control male carp (*cyp17a1*+/+;XY) with the same control female carp (*cyp17a1*+/+;XX), respectively (Figure 1A) [13]. All the carp were reared in the same pond at the Modern Ecological Fishery Research Base (Institute of Hydrobiology, Chinese Academy of Sciences, Wuhan, China). Then, the fish were transferred to an indoor culture system and housed in a recirculating system at 28 ± 0.5 °C, 14 h light/10 h dark photoperiod.

### 4.2. DNA Extraction, Genotype and Sex Confirmation

Fish tail clips were digested with HOME buffer (80 mmol∙L^−1^ EDTA, 100 mmol∙L^−1^ Tris pH 8.0, 0.5% SDS, Proteinase K 1 mg∙mL^−1^) at 65 °C for four hours. After digestion, sodium chloride and chloroform were subsequently added in a volume ratio of 1:1 and 1:0.6, respectively. After thorough mixing and centrifugation, the upper layer was precipitated with isopropanol, washed with 75% ethyl alcohol, and diluted with distillation-distillation H_2_O. The primers used for *cyp17a1* genotype and sex market are shown in Table 1.

### 4.3. Feeding Behavior Assay

The feeding behavior analysis was performed on the basis of our previous study [37]. The uniform-sized AF carp, control males and control females (0.43 ± 0.037 g, 1 month old) were randomly selected for the feeding behavior experiments, with three replicates per group, eight fish per replicate. After one week of acclimation, each fish was individually placed in a rectangular glass apparatus and acclimated for ten minutes before filming gegan (Figure 1B). Fish were fasted for 48 h before the diet was added. The video was recorded at 9:00 am with a camera (acA1920, 155uc NIR, Balser, Germany) at 90 frames/s. After 10 min, the diet was gently added, and the feeding behaviors of the carp were recorded for 10 min in consecutive days. The experiment lasted a total of 45 days and was repeated three times. After the feeding behaviors assay, fish were randomly selected and placed into the 100 mg/L MS-222 solution for anesthesia. The brains of nine carp per group were dissected after fasting for 48 h and feeding for 2 h, retained in liquid nitrogen and then kept at −80 °C.

### 4.4. Growth Performance, Feed Intake and Feed Efficiency

A total of 180 healthy common carp (15.19 ± 0.10 g, 3 months old) were stocked in nine 300-L fiberglass tanks (20 fish per tank) after 2 weeks of acclimation. They were fed to apparent satiation twice daily with a commercial pellet diet (protein: 31.0%; crude lipid: 3.0%; Fujian Tianma Science and Technology Group Co., Ltd., Fuqing, China). The feeding experiment lasted for 2 weeks. At the end of the experiment, fish were weighed to calculate body weight and weight gain (WG), feed intake (FI) and feed efficiency (FE).

### 4.5. qRT-PCR Analysis

RNA was isolated from the brain of common carp by extraction with TRIzol reagent (Invitrogen, Carlsbad, CA, USA). Specifically, 1 μg of RNA template was used for reverse transcription and cDNA synthesis using a first-strand cDNA synthesis kit (K1622, Thermo Scientific Fermentas, Waltham, MA, USA) according to the manufacturer’s instructions. Gene expression analysis was performed by qRT-PCR using the TransStart^®^ Tip Green qPCR SuperMix (AQ141-01, Transgen, Beijing, China) as described in our previous study [37]. All the experiments were performed in triplicate. The primers used in the present study are shown in Table 1. β-actin was used as the reference gene. Data were analyzed using a 2^−ΔΔCt^ method.

### 4.6. Data Analysis

The behavioral data were defined according to previous studies [22]. Fish behavior analysis was performed from the videos using an Ethovision XT version15 behavior analysis software (Noldus, Wageningen, The Netherlands). Data were analyzed by using SPSS 18.0 software and presented as mean ± standard error (SEM). All data were evaluated for their normality using the Kolmogorov–Smirnov test before the statistical analysis. Bartlett’s test was used to test for normality. The independent *t*-test was used to analyze differences between different groups. The Spearman correlation test was used to examine the correlations between different groups. Differences were considered significant at *p* < 0.05.

## Figures and Tables

**Table 1:** Primers used in PCR analysis.

Genes	Forward Primer (5′-3′)	Reverse Primer (5′-3′)
*gh*	TGCTATTGTCGGTGGT	CTGTCTGCGTTCCTCA
*npy*	TGCTTGGGAACTCTAACGGAA	GACCTTTTGCCATACCTCTGC
*agrp1*	CCGTGCATCCCTCATCAGC	GCTACGGCAGTAGCAGAAGGC
*orxin*	AATCCTGACGATGGGAAAGAG	TCGTGGTTTTAGCGACAAGTG
*β-actin*	GATGATGAAATTGCCGCACTG	ACCAACCATGACACCCTGATGT
*cyp17a1*	GTGGCATTGAAGAATTCGCAT	CCGTATTTCTTCTGCAGCTGC
Sex marker	GAGCATCCACTGTCAACTT	ACTCTTCCCAAACACTGATT

## Data Availability

The data presented in this study are available on request from the corresponding author.

## Supplementary Materials

The supporting information can be downloaded at: https://www.mdpi.com/article/10.3390/ijms252312517/s1.

## Abbreviations

AF: all-female; AGRP1, agouti-related neuropeptide 1; CRISPR/Cas9, Clustered Regularly Interspaced Short Palindromic Repeats/CRISPR-associated system; FE, feed efficiency; FI, feed intake; HCRT, orexin; GH, growth hormone; NPY, neuropeptide Y; SEM, standard error; WG, weight gain; WT, wild-type.
