# Supplementary material for: Characterization of Feeding Behaviors, Appetite Regulation and Growth Performance of All-Female (cyp17a1+/−;XX Genotype) Common Carp (Cyprinus carpio)
Source: Int J Mol Sci. 2024 Nov 21;25(23):12517. doi: 10.3390/ijms252312517 (PMC11641567; doi:10.3390/ijms252312517)
Supplement: Supplementary file 1 [file ijms-25-12517-s001.zip › ijms-3287272-supplementary/Supplementary figure.pdf]

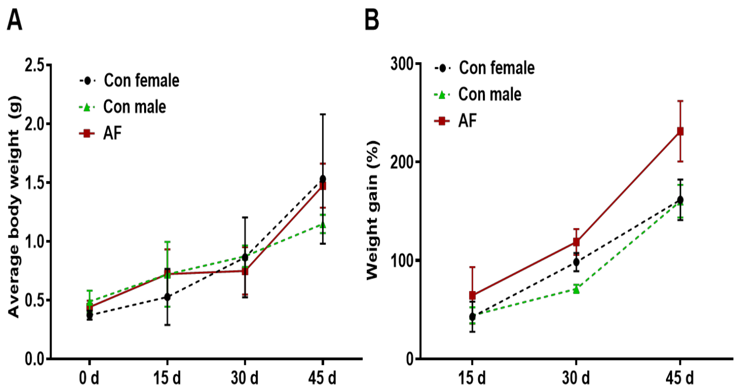

Supplementary Figure S1. The changes of the growth performance of AF and control common carp during feeding behaviors assay. (A) body weight. (B) Weight gain.
